# Review of the clinical status of cardiotoxicity of HER-2 positive breast cancer targeted therapeutic drugs

**DOI:** 10.3389/fonc.2024.1492203

**Published:** 2025-02-07

**Authors:** Xiang Zhang, Yulian Yin, Qiuting Yu, Xinlin Chen, Yiqin Cheng

**Affiliations:** ^1^ Department of Cardiology, Longhua Hospital Affiliated to Shanghai University of Traditional Chinese Medicine, Shanghai, China; ^2^ Department of Breast Surgery, Longhua Hospital Affiliated to Shanghai University of Traditional Chinese Medicine, Shanghai, China; ^3^ Hospital Administration Office, Shanghai University of Traditional Chinese Medicine, Shanghai, China

**Keywords:** breast cancer, HER-2, cardiotoxicity, targeted therapy, review

## Abstract

Breast cancer is a major health challenge for women worldwide, and human epidermal growth factor receptor 2 (HER-2)-positive breast cancers have a relatively high incidence and are highly aggressive. Targeted therapeutic agents, represented by trastuzumab, have been effective in improving the survival rate of HER-2-positive breast cancer patients. However, in clinical applications, this type of targeted drugs exhibits varying degrees of cardiotoxicity, and the mechanism of their cardiotoxicity is currently unclear. In this paper, we classify them into three categories: monoclonal antibodies (mAbs), small-molecule tyrosine kinase inhibitors (TKIs), and antibody-drug conjugate (ADCs). We list the evidence of cardiotoxicity for various drugs based on current clinical trials and summarize their corresponding epidemiological profiles. We also discuss the regulation of cardiotoxicity from three perspectives: clinical biomarkers of cardiotoxicity, permissive cardiotoxicity, and the current status of cardiotoxicity regulation.

## 1 Introduction

**Figure 1:**
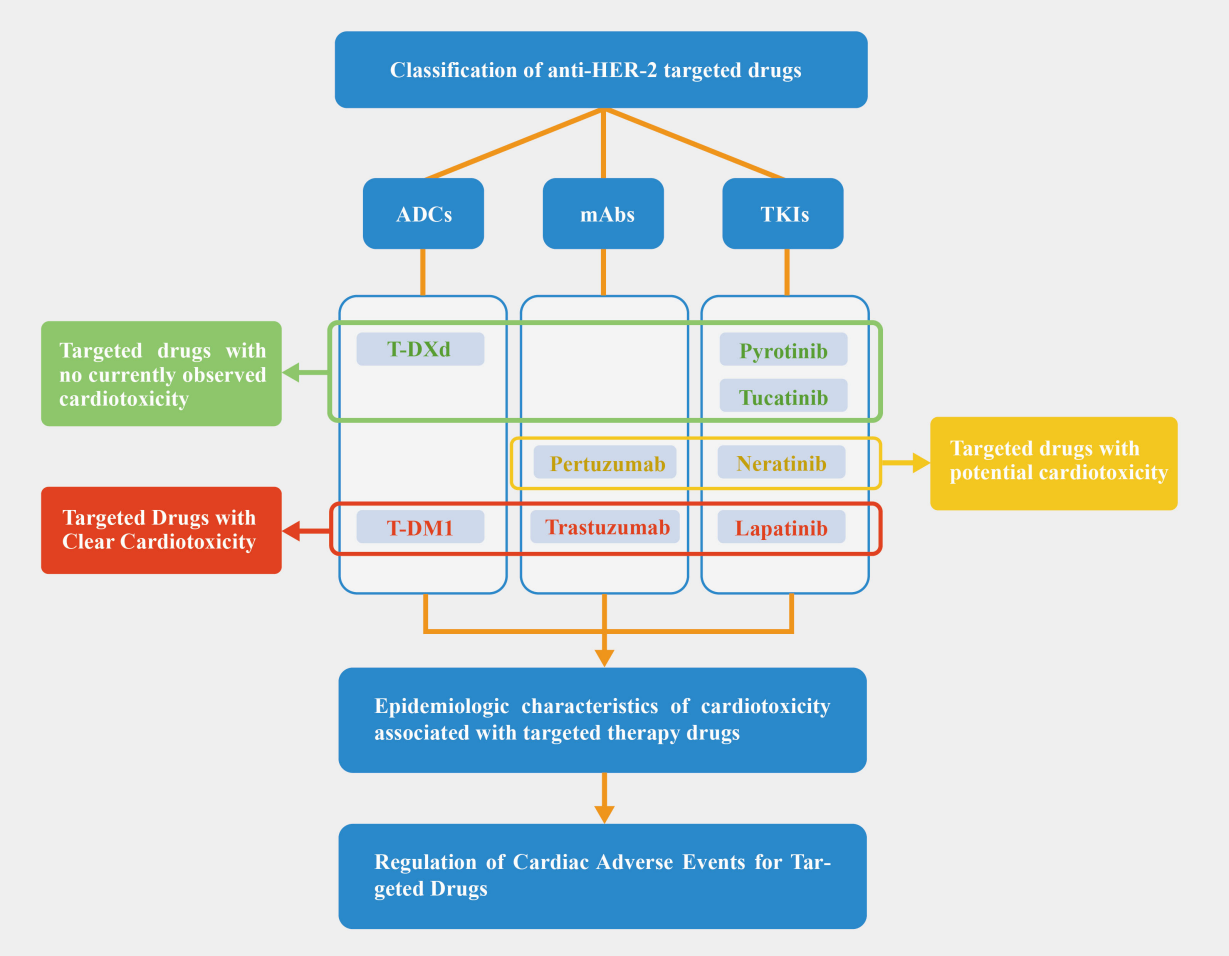
Article deconstruction chart. Based on the current clinical trial, we categorized targeted drugs into red, yellow, and green zones; Pertuzumab is not thought to increase cardiotoxicity when combined with trastuzumab, but some studies have pointed to an increase in cardiac risk, so we have placed it in the yellow zone.

Breast cancer is the most common cancer among women globally and in China, with over 2 million new cases reported in 2020, representing 11.7% of all new cancer diagnoses worldwide. In China, it ranks as the second most prevalent cancer, with an incidence of 45.29 cases per 100,000 people (1, 2). It is estimated that 20-25% of breast cancer patients have high expression of the human epidermal growth factor receptor 2 (HER-2) gene, and HER-2-positive breast cancer has the characteristics of strong invasion, easy recurrence, and poor prognosis compared to other subtypes of breast cancer (3). In recent years, the use of anti-HER-2 targeted drugs represented by trastuzumab has greatly improved the outcome of patients and increased survival (4). Currently, the anti-HER-2 targeted drugs used in clinical practice can be divided into three categories: monoclonal antibodies (mAbs), small-molecule tyrosine kinase inhibitors (TKI), and antibody-drug conjugates (ADCs). Some anti-HER-2 targeted drugs have shown cardiotoxicity that cannot be ignored in their application. This suggests that some patients were not able to complete the full course of HER-2 therapy. Cardiotoxicity has been observed during the treatment period and in the short-term follow-up after treatment for breast cancer. This includes effects such as decreased cardiac function, arrhythmias, and myocardial injury. Moreover, long-term tracking has shown an increased cardiovascular risk in breast cancer survivors 10-15 years after diagnosis (5). These individuals also experience a higher cardiovascular mortality rate within a 7-year period compared to the general population (6). This suggests late cardiotoxicity in breast cancer treatment. Therefore, evaluating the cardiac function of patients is critical when conducting targeted therapy. The aim of this paper is to review the clinical status, epidemiological characteristics, and regulatory status of anti-HER-targeted drugs based on relevant clinical reports. The general deconstruction of this paper is shown in **Figure 1**.

## 2 Classification of targeted drugs and current status of reports of their cardiotoxicity

HER-2 is a receptor tyrosine kinase that belongs to the epi-dermal growth factor receptor (EGFR) family. HER-2 has been found to be overexpressed in various malignancies including breast and gastric cancers (7). The overexpression of HER-2 may lead to the activation of HER-2 signal pathways which result in enhanced cellular proliferation, survival and reduced cell death (8, 9). Based on the different mechanisms of action, we have categorized the current targeted drugs against HER-2 into three groups: mAbs, TKIs, and ADCs.

### 2.1 Monoclonal antibodies

mAbs are the main type of targeted drugs used in clinical practice. The most widely used mAbs in breast cancer treatment include trastuzumab and pertuzumab.

Trastuzumab was approved by the food and drug administration (FDA) in 1998 as a combination therapy with paclitaxel for the treatment of HER-2 positive breast cancer. The mechanism of action of trastuzumab in anti-cancer therapy is that it binds to the extracellular, juxtamembrane portion of the HER-2 receptor and suppresses HER-2 signaling activity, resulting in the inhibition of downstream signaling pathways, cell cycle arrest, and a reduction in angiogenesis. Trastuzumab’s binding to the extracellular domain of HER-2 results in antibody-dependent cell-mediated cytotoxicity and prevents the cleavage of the HER-2 receptor’s extracellular domain (10). Trastuzumab significantly reduces recurrence and improves survival when used in the treatment of patients with HER-2 positive Breast Cancer (10). Now, trastuzumab can be used not only in the treatment of breast cancer, but also in the targeted treatment of gastric cancer (7) and colorectal cancer (11). Trastuzumab has been observed in a large number of clinical trials with definite cardiotoxicity, the epidemiologic features of which will be discussed in detail in Part III. The mechanism of trastuzumab cardiotoxicity has not yet been definitively described, and the following is a possible explanation. HER-2 and other members of the epidermal growth factor receptor family, including HER-1, HER-3, and HER-4, are also expressed in cardiomyocytes. This is thought to be the physiologic basis for the cardiotoxicity of trastuzumab (9). It is thought that trastuzumab binds to HER-2 in cardiomyocytes, preventing it from interacting with other receptors and thus inducing stress. The induction of stress on cardiomyocytes produces reactive oxygen species, which in turn leads to cardiac insufficiency and apoptosis of cardiomyocytes (12). However, there are some problems with this explanation, such as the inability to explain the reversibility of trastuzumab cardiotoxicity (9). The field throws the need for more research.

Recent research has clarified the interaction between anthracycline drugs and trastuzumab, explaining why they are often used sequentially due to increased cardiac toxicity when given together (13). It is believed that the use of trastuzumab interferes with the repair of myocardial cells influenced by anthracycline drugs (14). Anthracycline chemotherapy drugs induce oxidative stress damage to myocardial cells by disrupting the normal cycling mechanism of topoisomerase-2β. In the absence of trastuzumab, damaged myocardial cells would either undergo cell death or initiate the cell repair process. Trastuzumab interferes with cell repair, leading to a greater proportion of damaged or dead myocardial cells. If trastuzumab is used during this sensitive period following anthracycline drug administration, a larger proportion of myocardial cells will undergo cell death. Retrospective analysis of clinical trials has confirmed this explanation: Michael S. Ewer et al. found that the shorter the time interval between initiating anthracycline drugs and trastuzumab, the higher the incidence of cardiac toxicity (15).

Pertuzumab was approved by the FDA in 2012 and China’s National Medical Products Administration in 2018 for the same indication. Pertuzumab is directed against the extracellular dimerization domain of HER-2. Pertuzumab is believed to prevent HER-2 interactions with other members of the EGFR family such as HER-1, HER-3 and HER-4. This results in the inhibition of proliferation and survival signals emanating from these receptors (9). The combination of trastuzumab and pertuzumab is commonly used to treat HER-2 positive breast cancer. However, the combination has been associated with a significant increase in skin-related adverse events, but it does not increase the risk of cardiovascular toxicity (16, 17). Analysis of clinical trials conducted prior to 2013 by Antonis Valachis showed that the overall incidence of Congestive heart failure (CHF) and decreased left ventricular ejection fraction (LVEF) were 0.88% and 3.1% for combination therapy and 1.49% and 2.9% for single-drug therapy with trastuzumab, respectively (18). This conclusion was confirmed in a 5-year follow-up study by Gianni et al. (19), and subsequent large clinical trials and meta-analyses confirmed that the combination of trastuzumab and pertuzumab has high therapeutic benefits without increasing the risk of cardiovascular toxicity. Multiple large-scale clinical trials and meta-analyses conducted later have confirmed that the combination of the two treatments provides greater benefits in cancer treatment and does not increase the risk of heart toxicity. The increased adverse events are mostly tolerable events such as rash and diarrhea (20–25). Therefore, the 2021 China HER-2 Positive Breast Cancer Expert Consensus states that patients who are suitable for single-targeted treatment can consider starting treatment with trastuzumab and pertuzumab (26). However, the absence of increased cardiac burden in combination with trastuzumab does not mean that there is absolutely no cardiotoxicity. Mayuko Ito et al. reported on 2 Japanese patients who did not develop cardiotoxicity on trastuzumab treatment, then received pertuzumab due to the development of metastases, and developed left heart insufficiency during treatment (27). Alhussein MM et al. conducted a meta-analysis of 8 RCTs, which include gastroesophageal trial and seven breast cancer trials. In these trials, all patients were treated with trastuzumab except for pertuzumab as well as placebo. The analysis showed that pertuzumab increased the risk of clinical heart failure (HF) (RR [95% CI]: 1.97 [1.05-3.70]; I2 = 0%) but not left ventricular systolic dysfunction (RR [95% CI]: 1.19 [0.89-1.61]; I2 = 19%) compared with placebo (28).

### 2.2 Tyrosine kinase inhibitors

TKIs are small molecules that bind competitively to the EGFR family’s binding domains, blocking HER-2 signaling and inhibiting phosphorylation to exert antitumor effects. They can cross the blood-brain barrier, making them effective in metastatic breast cancer (29). Key TKIs include lapatinib, neratinib, tucatinib, and pyrotinib. Neratinib is FDA-approved for early-stage breast cancer, while the others are used for advanced or metastatic cases. Lapatinib has the most reports of cardiac toxicity, while neratinib has few, and tucatinib and pyrotinib show no clear evidence of cardiac toxicity

Lapatinib was approved by the FDA in 2007 and approved by China in 2013. It is mainly used in combination with capecitabine for HER-2 overexpressed metastatic or advanced breast cancer patients who have received previous HER-2 treatment. Lapatinib’s mechanism involves reversible inhibition of EGFR and HER-2, along with downstream signaling pathways inhibition. Lapatinib blocks phosphorylation of the tyrosine kinase residues inhibiting cell proliferation by blocking the mitogen-activated protein kinase and phosphoinositide 3-kinase pathways (30). Although lapatinib-related cardiotoxicity was the most reported of all TKIs used in breast cancer, its most prominent adverse effects were actually rash and diarrhea (30). Since cardiotoxicity is not a major adverse effect, the mechanisms involved are less well studied. Some studies suggest that it is similar to trastuzumab, which inhibits the action of HER-2 in cardiomyocytes, leading to decreased myocardial contractility as well as apoptosis (31). We will discuss lapatinib-associated cardiotoxicity in more detail in Section 3.

Neratinib was approved by the FDA in 2017 and is currently the only TKI-type drug approved for use in early-stage HER-2 positive breast cancer. Neratinib is a noncompetitive TKI of adenosine triphosphate. Neratinib inhibits EGFR/HER-2 activity by covalently binding to the cysteine residue at position 805 in the kinase domain of these receptors, resulting in the inhibition of kinase activity (32). The most common side effects are diarrhea and nausea (33). Not all clinical trials observed cardiac adverse events (34). This is why some researchers believe neratinib is not associated with this toxicity (30). In the Phase III NALA trial, cardiac events(CE) such as arrhythmias (3.3%), ischemic heart disease (0.7%), and decreased LVEF (4.3%) occurred in the neratinib combined with capecitabine treatment group. The Phase III NALA trial, which also compared the clinical efficacy of neratinib and lapatinib, found that neratinib use does not raise new concerns about cardiac safety and that its cardiac toxicity is at the same level or lower than lapatinib (35). Further large-scale clinical trials or clinical observations are needed to determine the exact cardiac toxicity and manifestation.

Tucatinib is a highly selective and reversible HER-2 inhibitor approved by the FDA in 2017 for HER-2 positive breast cancer with brain metastases (36). Tucatinib inhibits the protein tyros-ine kinase activity of HER-2 and exerts minimal inhibition of EGFR (37). The main side effects of Tucatinib are diarrhea, hand-foot syndrome, and nausea (38). Currently, there has been no observation of cardiac toxicity associated with Tucatinib (38, 39).

Pyrotinib is a non-reversible HER-2 inhibitor that was approved for late-stage and metastatic breast cancer in China in 2018. Pyrotinib can inhibit the autophosphorylation by covalently binding to ATP binding sites in intracellular kinases of HER-1, HER-2 and HER-4, thus blocking the activation of downstream signaling pathways to inhibit tumor cell growth (40). Its main side effects are similar to those of Tucatinib, including diarrhea and hand-foot syndrome. However, clinical observation of neutropenia as an adverse reaction has been observed with Pyrotinib. Currently, there has been no observation of cardiac-related adverse reactions associated with Pyrotinib (41).

### 2.3 Antibody-drug conjugates

ADCs are medications that link an antibody targeting tumor antigens with a cytotoxic chemotherapy drug. They work by the antibody mediating cell entry, where the cytotoxic drug is released to kill tumor cells. Compared to traditional targeted and cytotoxic drugs, ADCs offer higher selectivity and lower toxicity, showing great promise (42). Currently, widely used ADCs in breast cancer include Trastuzumab Emtansine (T-DM1) and Trastuzumab Deruxtecan (T-DXd).

T-DM1 is the first approved ADCs drug, which is a combination of trastuzumab and microtubule inhibitor emtansine (DM1), and was approved by FDA in 2013 for the treatment of HER-2 positive metastatic breast cancer patients who failed trastuzumab treatment. T-DM1 should not be considered a substitute for trastuzumab, but more of a bifunctional reagent. “In tumor treatments, along with the role of trastuzumab, DM1 are also internalized along with their receptors. After internalization, it is believed to undergo lysosomal degradation and that results in the release of the microtubule inhibitor drug that induces cell cycle arrest and cell death (9). The TH3RESA trial showed that T-DM1 significantly improved the prognosis of patients who had failed two prior HER-2 treatments (43). In early clinical trials, T-DM1 showed mild and reversible toxicity in patients (44). In many phase-I clinical trials, the common adverse reactions of T-DM1 were thrombocytopenia, transaminase elevation, fatigue, but no obvious cardiac toxicity was observed (43–45). However, with the increasing clinical application and more clinical trials, more cardiac toxicity events were observed. The cardiac toxicity and related manifestations of T-DM1 will be described in detail in Section 3. Given that T-DM1 still binds HER-2 receptors with trastuzumab, the mechanism of cardiotoxicity may be the same as with trastuzumab.

T-DXd, a new ADC drug, is composed of trastuzumab and type I topoisomerase inhibitor (DXd, a derivative of the camptothecin analogue exatecan), and was approved by FDA in 2019 for breast cancer patients who have received ≥2 HER-2 treatments (46). Similar to T-DM1, T-DXd will release DXd linked to trastuzumab upon binding to the HER-2 receptor via trastuzumab. DXd binds to and inhibits topoisomerase I-DNA complexes, leading to inhibition of DNA replication, cell cycle arrest and tumor cell apoptosis (46). Due to the late approval, there are few clinical trials related to T-DXd. A retrospective study in 2017 indicated that the main adverse reactions were bone marrow suppression, however, the study only involved 24 patients (47). In 2022, a phase III trial of late-stage breast cancer patients with low expression of HER-2 conducted by Shanu Modi et al. (48) showed that the most common adverse reactions of T-DXd were nausea, fatigue, and alopecia. The most common grade 3 or higher adverse reactions were neutropenia and anemia, and 12.1% of the patients had drug-related pulmonary disease or pneumonia. So far, T-DXd has shown more pulmonary toxicity than cardiac toxicity (49). But considering its short time on the market, whether it has cardiac toxicity still needs further observation.

### 2.4 The “multiple-hit” hypothesis

One challenge in understanding the mechanism of cardiotoxicity related to targeted drugs is connecting microscopic studies with the macroscopic manifestation of cardiotoxicity, especially given its selective and reversible nature. “Selective” means cardiotoxicity occurs in only some patients, while “reversible” refers to type II cardiotoxicity caused by targeted drugs, such as monoclonal antibodies like trastuzumab and TKIs (50). Unlike Type I cardiotoxicity caused by anthracyclines, type II cardiotoxicity has the potential for recovery after damage.

This phenomenon may be explained by the “multiple-hit hypothesis” introduced by Lee W. Jones et al. in 2006 (51). Their hypothesis seeks to elucidate the occurrence of cardiotoxicity in breast cancer patients. Patients diagnosed with breast cancer often display different levels of cardiovascular risk, which can be further exacerbated by the varying degrees of cardiovascular damage induced by adjuvant therapies. These cumulative exposures play a significant role in the eventual development of cardiovascular diseases.

By examining the interaction between anthracyclines and trastuzumab, we apply this hypothesis to Type II cardiotoxicity, introducing the concept of damage to fragile myocardial cells. While normal cells may recover from targeted drug-induced cardiotoxicity, “fragile” cells are more prone to death or slower repair. This may explain why some studies observe cell death or Type I cardiotoxicity with drugs associated with type II cardiotoxicity. Further research is needed to validate this hypothesis

We classified the drugs into three categories based on cardiotoxicity: (a) Clear cardiotoxicity: trastuzumab, lapatinib, and T-DM1, with well-documented effects; (b) Potential cardiotoxicity: pertuzumab and neratinib, where effects have been observed but require further investigation; (c) No observed cardiotoxicity: tucatinib, pyrotinib, and T-DXd, though this doesn’t guarantee complete safety. **Figure 1** uses red, yellow, and green to differentiate these categories. As anti-HER-2 drugs are increasingly used, cardiac adverse events are more frequently reported. **Table 1** summarizes study durations, cardiotoxic manifestations, and mechanisms. Drugs with confirmed cardiotoxicity, such as trastuzumab, lapatinib, and T-DM1, are discussed in Section 3.

**Table 1:** Mechanisms of cardiotoxicity and clinical manifestations of some HER-2-targeted drugs.

Classification	Drug Name	Mechanism of action	Time of Approved Clinical Application	Cardiotoxicity	Clinical manifestations of cardiotoxicity
mAbs	Trastuzumab	Immune-mediated response causing internalization and downregulation of HER-2 (10)	1998	Clear	Symptoms of CHF, Decrease in LVEF, Arrhythmia, Coronary calcification (52–54)
	Pertuzumab	Prevents HER-2&HER-3 dimerization (combined with trastuzumab and docetaxel) (9)	2012	Potential	Symptoms of CHF, Decrease in LVEF (28)
TKIs	Lapatinib	Blocks EGFR/HER-2 protein kinase activity (30)	2007	Clear	Symptoms of CHF, Decrease in LVEF (55, 56)
	Neratinib	Blocks EGFR/HER-2 activity by covalently binding with a cysteine side chain in those receptors (32)	2017	Potential	Decrease in LVEF, Arrhythmia, Ischemic heart disease (35)
	Tucatinib	Highly selective inhibitor of the kinase domain of HER-2 receptor (37)	2017	Not yet	
	Pyrotinib	Blocks HER-1&HER-2&HER-4 activity by covalently binding with ATP binding sites of intracellular kinase regions (40)	2018	Not yet	
ADCs	T-DM1	Trastuzumab and DM1 (cytotoxic maytansinoid);DMI binds microtubules and inhibits cell division in the tumor cells (9)	2013	Clear	Symptoms of CHF, Decrease in LVEF, Arrhythmia, Myocardial ischemia (57)
	T-DXd	Trastuzumab and a topoisomerase I inhibitor conjugate deruxtecan (46)	2019	Not yet	

## 3 Epidemiologic characteristics of cardiotoxicity associated with targeted therapy drugs

Cardiotoxicity induced by cancer treatment can manifest as arrhythmia, arterial hypertension, thrombus embolism, angina, myocardial infarction or HF (52). Studies have found that cardiotoxicity associated with trastuzumab mainly involves HF, but may also involve coronary calcification (53) and arrhythmia (54). However, the incidence and related research of the latter two are relatively rare, especially coronary calcification. Different definitions of cardiotoxicity lead to differences in cardiotoxicity incidence calculations and results across studies. Based on the definition above, the author further searched the matched literature to summarize the probability of occurrence, time of occurrence, reversibility, and risk factors of CE associated with breast cancer treatment with trastuzumab, lapatinib, and T-DM1, and the contents are presented in **Table 2**.

**Table 2:** Cardiotoxicity epidemiological characteristics of different types of targeted therapies.

Drug	Treatment	Occurrence probability	Reversibility	Risk Factors
Trastuzumab	Paclitaxel for 12 weeks with trastuzumab, and trastuzumab was continued for 1 year	Symptomatic CHF responsive to intervention (0.5%); Significant asymptomatic LVEF decline (3.2%) (65).	Reversible or partially reversible	Previous CVD^a^, Hypertension^b^, Diabetes mellitus^c^, Chronic kidney disease ^d^, Age, Current smoker or significant smoking history, LVEF, cTn and NPs elevated at baseline measurements, anthracyclines and RT to left chest or mediastinum (84–96)
	Doxorubicin and cyclophosphamide (AC) followed by paclitaxel plus trastuzumab followed by trastuzumab alone	LVEF decrease to below the LLN (4.7%); LVEF decrease by >10 points from baseline (22.6%) (66).		
	Primary therapy (including, surgery, chemotherapy, and radiotherapy as indicated) and receive trastuzumab for 1 year	Primary cardiac endpoint^e^ (1%); Secondary cardiac endpoint^f^ (4.4%) (67).		
	Able to accept trastuzumab treatment, no other requirements	Symptomatic CHF (2.8%);significant drop in LVEF^g^ (7.6%) (68).		
Lapatinib	A meta-analysis involving multiple clinical studies	The sum of all cardiac events (3.0%); LVEF decrease (1.8%) (63).	Reversible or partially reversible	Not available
T-DM1	A meta-analysis involving multiple clinical studies	The sum of all cardiac events (3.37%) (57).	Reversible or partially reversible	LVEF was less than 55% before treatment, Age (>65) (57)

a coronary heart disease, myocardial infarction, heart failure, cardiomyopathy, severe heart valve disease, arrhythmia; ^b^Systolic BP >140mmg Hg or diastolic BP >90mm Hg, or on treatment; ^c^HbA1c >7.0% or >53mmol/mol or on treatment; ^d^Estimated glomerular filtration rate <60ml/min/1.73m^2^; ^e^NYHA class III or IV toxicity, confirmed by a cardiologist, and a clinically significant LVEF drop of at least 10 percentage points from baseline and to an absolute LVEF below 50%, or cardiac death; ^f^asymptomatic (NYHA class I) or mildly symptomatic (NYHA class II) with a clinically significant LVEF drop of at least 10 percentage points from baseline and to an absolute LVEF below 50% confirmed by repeat assessment; ^g^at least one drop in LVEF of≥ 10 percentage points from baseline to < 50%.

### 3.1 Clinical manifestations of cardiotoxicity of various targeted drugs

Report of trastuzumab about its adverse CE mainly focusing on CHF, and has a relatively mature definition and diagnostic criteria. Its main manifestations are a decrease in LVEF, a decrease in cardiac function, and the appearance of symptoms and signs related to CHF. Notably, a decrease in LVEF is often not accompanied by discernible symptoms, making it a challenging clinical diagnosis. At present, it is generally believed that asymptomatic LVEF decreased by 10% compared to the baseline state, which can be considered CE (58). Some studies also suggest that asymptomatic LVEF decreases by 10% or less than baseline and is below the institution normal value (LLN), which can be considered CE (59–61). Regarding the decline in cardiac function grading, New York grading is often used. Symptoms and signs related to CHF can be manifested as shortness of breath, and chest tightness (62).

In early studies, lapatinib-related CE were defined as symptomatic CHF or asymptomatic reduction of LVEF (more than 20% or less than LLN) (55). With the continuous popularization and application of lapatinib, the definition of adverse CE has also expanded. On the original basis, arrhythmia events related to lapatinib were observed, most of which were QT interval prolongation (56). However, compared with the decrease in LVEF, the probability of arrhythmia is extremely low. In a meta-analysis study involving 6646 participants in 45 studies (63), 621 patients were found to have adverse CE, and only 3 patients had arrhythmia, of which only 1 was related to breast cancer.

The Phase I trial of T-DM1 did not reveal substantial cardiotoxicity; however, subsequent Phase II and III studies confirmed its cardiotoxic effects. Notably, a significant decrease in left ventricular ejection fraction (LVEF) was observed in 1.7% of patients treated with T-DM1 in a large multi-center Phase III study (64). Furthermore, a meta-analysis conducted in 2020, which encompassed 1961 patients, highlighted various CE associated with T-DM1, including CHF symptoms, reduced LVEF, arrhythmia, and myocardial ischemia (57).

### 3.2 Probability of occurrence

There are divergent views on the probability of adverse CE after the use of trastuzumab. The literature indicates that the proportion of patients with asymptomatic LVEF decline fluctuates between 3.2% and 22.6%, and the incidence of symptomatic CHF ranges from 0.5% to 2.8% (65–68). A large meta-analysis involving more than 29,000 patients and 58 experiments noted (69) that the cardiotoxic events in the study could be divided into severe cardiotoxicity and mild or asymptomatic cardiotoxic events, and the difference in probability of occurrence was mainly concentrated on mild or asymptomatic cardiotoxicity. The probability of severe cardiotoxicity is about 3%, while the probability of mild or asymptomatic cardiotoxicity ranges from 1% to 80% (69). The difference in the probability of mild or asymptomatic cardiotoxicity may be related to the lack of clear and uniform diagnostic criteria and the failure of effective data in mild cases to be well monitored. The study also pointed out that the probability of cardiotoxicity in early breast cancer (EBC) and metastatic breast cancer (MBC) was also different, and the probability of cardiac adverse events in MBC was higher, which indicated that we should strengthen the monitoring of cardiac indicators for patients with MBC. It has been pointed out by multiple researchers that the probability of cardiac adverse events observed in Randomized Controlled Trials (RCT) is lower than that observed in observational studies. Therefore, the probability of cardiotoxicity induced by trastuzumab may be underestimated (69–71).

The cardiac risk of lapatinib is relatively small compared with that of trastuzumab. Some scholars in earlier relevant studies believed that the cardiac toxicity risk of lapatinib was < 2% (72). In a 43-item meta-analysis of lapatinib (55), researchers found that after patients received an average of 13.7 weeks of treatment at a dose of 1000–1500 mg/day, 1.6% of patients developed CE, and 0.2% of patients developed symptomatic HF. Most of the cardiotoxic events occurred within 9 weeks (59%), but 14% of cardiotoxic events occurred after 25 weeks of dosing. However, the study included multiple cancers and data on breast cancer patients were not listed separately. The risk of cardiotoxicity observed in subsequent meta-analyses was also increased compared to earlier periods, with an overall incidence of cardiac adverse events of 2.7%. The total incidence of cardiac adverse events in breast cancer patients is 3%, slightly higher than the incidence of all types of cancer patients (63).

In a large, multi-center Phase III study (64), the probability of a decreased LVEF in patients using T-DM1 was 1.7%. In a 2020 meta-analysis involving 196 patients, it was pointed out (57) that the incidence of CE was 3.37%, and the decline in LVEF had the highest probability, 2.04%. There is less clinical data on ADC-related cardiotoxicity and more long-term updated research evidence is needed.

When analyzing the frequency of occurrence, it should also be noted that the combination of drugs has an effect on the heart. For example, trastuzumab in combination with pertuzumab, which is by far the most common, has been shown in trials not to additionally increase the risk of cardiotoxicity, as mentioned in the previous section. However, as newer drugs are rolled out in the clinic, more combinations will emerge. In the NeoALTTO trial, Baselga J et al. compared the efficacy of combining lapatinib with trastuzumab versus using lapatinib or trastuzumab alone. Significant cardiotoxic events were not observed in any of the three groups (73). A meta-analysis by Rahmani H et al. showed that the combination of trastuzumab and lapatinib did not increase the risk of a decrease in LVEF compared with the combination of trastuzumab and pertuzumab, in patients with normal cardiac function prior to the start of therapy (74).

Another factor that affects the incidence of cardiotoxicity is the nature of the study. Researchers found a higher incidence of cardiotoxicity in observational studies compared to RCTs, providing quantitative descriptions: EBC had incidences of 1.7% in RCTs versus 3.2% in observational studies, while MBC showed incidences of 2.8% in RCTs versus 4.4% in observational studies (69). We believe this difference comes from the strict inclusion criteria for patients in RCT. For example, in the large clinical trial on trastuzumab conducted by Piccart-Gebhart MJ et al. (75), patients were asked to have a normal LVEF despite chemotherapy and radiotherapy. Patients with a previous history of CHF, myocardial infarction, and angina requiring medication were also excluded from the trial. In the clinical trials related to lapatinib and T-DM1, we all saw a requirement for LVEF in the inclusion criteria and exclusion of some cardiac patients in the exclusion criteria (64, 76). Therefore, we speculate that the nature of this study affects the incidence of cardiotoxicity in a way that is generalizable. The incidence of cardiotoxicity reported in the RCT may be lower than what is observed in real-world data.

### 3.3 Application time and reversibility

At present, the cardiotoxicity of anti-HER-2 drugs is considered related to their application time, and long-term treatment will bring higher cardiac risk. However, no consensus has been reached among researchers on cumulative use risk. Some researchers believe that the second year of cumulative trastuzumab use is associated with the highest peak of adverse CE (69). Conversely, some researchers believe that the risk increases linearly with each year of use, with an approximate annual risk increase of 1% after the second year (71). Currently, a one-year treatment with trastuzumab is commonly used. In order to reduce heart-related risks, some researchers began experimenting with trastuzumab for six months to further reduce the incidence of cardiac adverse events. A multi-center clinical trial involving 3,380 patients, conducted by Xavier Pivot et al. (77) in France, concluded that while a shorter treatment cycle could reduce heart-related risks, it would also diminish the effectiveness of tumor treatment. Consequently, the 12-month treatment cycle was discarded as a treatment option. However, according to the multi-center clinical trial involving 2,045 patients conducted by Helen Earl et al. (78), the related benefit of the six-month treatment cycle is not worse than the 12-month treatment cycle, and at the same time, it will reduce heart-related risks. Therefore, it is suggested to shorten the treatment cycle of trastuzumab in clinical practice. Contradictory results suggest that more evidence is still needed in this field to support the related conclusions, and balancing tumor benefit and cardiotoxicity remains one of the clinical challenges.

Cardiotoxicity caused by trastuzumab is generally considered reversible. In some studies, symptom relief and even LVEF increase are observed in patients after drug discontinuation (79), and even patients in some studies who re-enter the experimental group after cardiac function recovery are treated with trastuzumab (80). Not all patients with trastuzumab-associated cardiotoxicity show reversible effects, and those with improved LVEF may not return to baseline. The factors predicting reversibility remain unclear.

Similar to trastuzumab, the current study has pointed out that the cardiotoxicity of lapatinib is mostly reversible, and most patients with cardiotoxic events can recover or partially recover heart function after drug withdrawal (81). There are few studies on whether T-DM1 cardiotoxicity is reversible, but only literature reports so far. Most of the LVEF decreases observed in relevant experiments are reversible (64).

### 3.4 Time of occurrence of cardiotoxic events

Cardiotoxic events associated with anti-HER-2 targeted agents may occur during or after completion of therapy, but most occur during treatment. In a real-world study involving 160 patients, the onset time of heart-related trastuzumab adverse reactions was 28.5 weeks (82). In another retrospective study involving 265 patients (79), the majority of LVEF decreases occurred within six months after the start of treatment. However, there is no large-scale epidemiology revealing the time point of onset of trastuzumab cardiotoxicity.

Some long-term follow-up results also support this observation. Piccart M et al. followed breast cancer patients treated with trastuzumab and pertuzumab. Among 18 patients who experienced a primary CE and 65 who had a secondary CE, only one event in each group occurred during an additional 2.5 years of follow-up after treatment ended (83). Cameron D et al. followed breast cancer patients treated with trastuzumab for either one or two years. The study showed that the cumulative incidence of primary and secondary CE did not increase for a period after the end of treatment (approximately 1 year for patients treated for one year, and 2-3 years for those treated for two years) (67).

But several recent studies have challenged this finding, and the intersection of breast cancer and cardiovascular disease (CVD) may extend beyond the short-term follow-up period during and after treatment. Bradshaw PT et al. found that CVD-related mortality among breast cancer survivors increased significantly after 5 years, with the risk of death from CVD at 7 years nearly double that of non-breast cancer controls (6). Similarly, Koric A et al. observed that breast cancer survivors more than 10 years post-diagnosis had a 33% higher risk of circulatory disease and a 19% higher risk of heart disease (5). Unfortunately, neither study analyzed subgroups of patients receiving targeted therapy, but it still reveals that there is also a distinction between early and late cardiotoxicity of targeted drugs. More long-term studies on cardiotoxic events associated with anti-HER-2 targeted therapy are still needed.

### 3.5 Relevant risk factors

There are many risk factors associated with trastuzumab, which we have divided into two categories: those related to the patient’s own condition; and those related to other oncology treatments received. The first category, which has now been confirmed by research, includes: Previous CVD (coronary heart disease, myocardial infarction, HF, cardiomyopathy, severe heart valve disease, arrhythmia), Hypertension (Systolic blood pressure (BP) >140mmg Hg or diastolic BP >90mm Hg, or on treatment), Diabetes mellitus (HbA1c >7.0% or >53mmol/mol or on treatment), Chronic kidney disease (Estimated glomerular filtration rate <60ml/min/1.73m^2^), Age, Current smoker or significant smoking history (84–89). Elevated biomarker baseline compared to normal values found prior to treatment is also a risk factor, includes: LVEF, Cardiac troponins (cTn) and Natriuretic Peptides(NPs). Serum biomarkers (90–93). The second group of risk factors includes anthracyclines and radiotherapy (RT) to left chest or mediastinum (94–96). Anthracyclines, as chemotherapeutic agents, are inherently cardiotoxic, and their use as risk factors for cardiotoxicity in trastuzumab therapy is well recognized (94). This is because in clinical trials, the incidence of CE was found to be “unacceptable” when both were used together (97). In the trial (98), anthracyclines combined with trastuzumab were associated with the highest percentage of CEs (27%) and the highest percentage of serious CEs (16%). Some of the risk factors are dual risk factors for the use of anthracyclines and trastuzumab. Breast cancer patients with dual risk factors who are treated with anthracyclines followed by trastuzumab should receive intensive monitoring. To make it easier for clinicians to screen these populations, we have listed dual risk factors: Previous CVD (coronary heart disease, myocardial infarction, HF, cardiomyopathy, severe heart valve disease), Hypertension, Diabetes mellitus, Chronic kidney disease, Age, Current smoker or significant smoking history, LVEF, cTn and NPs elevated at baseline measurements, anthracycline, RT to left chest or mediastinum. The debate about radiotherapy as a risk factor remains, with some studies suggesting that radiotherapy, either left or right, does not increase the rate of CE during trastuzumab treatment (99, 100). Therefore, unlike anthracyclines, more research is needed on the effect of radiotherapy on trastuzumab cardiotoxicity.

There are fewer relevant similar studies for lapatinib and T-DM1.Analysis has pointed out that age greater than 65 years old and LVEF less than 55% before the start of treatment are the risk factors for T-DM1 treatment (57). However, in a retrospective analysis of ATEMPT conducted in 2022, it was considered that there was no direct and inevitable connection between the occurrence of baseline LVEF and CE (101). Therefore, there is still a lack of a unified understanding of the risk factors for T-DM1 cardiotoxicity. The lack of cardiotoxic risk factor analysis of lapatinib may be due to the fact that the most common adverse effects are not associated with cardiotoxicity.

In summary, a certain proportion of cardiotoxic events occur with three drugs, and their occurrence probability also varies, which may be related to the popularity of drugs, adaptation to the population, application and treatment course, etc. In addition, data for patients with mild or asymptomatic cardiotoxicity deserve more attention.

## 4 Regulation of cardiotoxicity for targeted drugs

Clinicians treating HER-2-positive breast cancer must manage the cardiotoxicity risks of targeted therapies. We will discuss the regulation of targeted drug cardiotoxicity from three perspectives: cardiotoxicity clinical biomarkers, permissive cardiotoxicity, and the current status of cardiotoxicity regulation.

### 4.1 Cardiotoxicity clinical biomarkers

The discussion of clinical biomarkers should begin with Transthoracic Echocardiogram (TTE), particularly the use of LVEF, which is the most common metric for cardiotoxicity monitoring. LVEF is a well-established diagnostic and predictive marker for cardiotoxicity and is often used as a primary or secondary endpoint in clinical trials (67, 102). However, LVEF is more commonly used for diagnosis rather than prediction. Recently, global longitudinal strain (GLS) from TTE has gained attention for its higher sensitivity in detecting early cardiotoxicity and predicting subsequent LVEF reduction (103, 104).

In addition to TTE, traditional cardiac serum biomarkers like cTn and NPs are crucial for detecting cardiac damage and are key in monitoring cardiotoxicity in patients undergoing cardiotoxic therapies. Cardiac troponins, particularly Cardiac troponin T (cTnT) and Cardiac troponin I (cTnI), are vital in this regard (102). A retrospective analysis by Li Zhang et al. of 420 patients treated with trastuzumab or its combination with pertuzumab showed that cTnI trends were similar to LVEF, with a significant predictive capacity for cardiotoxicity (AUC = 0.724, P < 0.05) (105). Cardinale D et al. also demonstrated that elevated cTnI predicts trastuzumab-induced cardiotoxicity and is linked to poor LVEF recovery, with cTnT showing similar or even greater sensitivity (96). cTnT was equally predictive of trastuzumab-induced cardiotoxicity (106). Even in some cases, cTnT is more sensitive than cTnI (102). However, cTn’s predictive role in trastuzumab-related cardiotoxicity does not extend to all targeted agents. For example, Morris PG et al. found that while cTnI changes may precede LVEF reductions in patients on trastuzumab and lapatinib, they do not predict the onset of CHF (107).

NPs include brain natriuretic peptide (BNP) and N-terminal part of the pro-peptide of BNP (NT-proBNP). BNP and NT-proBNP are among the most commonly used biomarkers for the diagnosis and prediction of HF, and NT-proBNP is generally considered to be more sensitive than BNP (108). Andersson AE et al. found in a study of 136 patients that an increase in NT-proBNP was indicative of the onset of cardiotoxicity. An NT-proBNP increase of 75.8 pg/mL from baseline showed 100% sensitivity and 95% specificity in detecting CEs. They concluded that analyzing the change in NT-proBNP values during trastuzumab treatment in patients can replace cardiac ultrasound for monitoring CEs. If their research can be validated and replicated, it will save patients and the healthcare system money and time. Zardavas D et al. investigated the predictive role of cTnT, cTnI, and NT-proBNP for trastuzumab cardiotoxicity and showed that unlike cTnT, cTnI, baseline levels of NT-proBNP did not predict CE (109). The difference in conclusions between the two studies reveals that, unlike cardiac troponin, more evidence is still needed for NPs as clinical biomarkers.

In addition to the traditional cardiac biomarkers such as LVEF, cTn and NPs, there are also emerging biomarkers such as Single nucleotide polymorphisms, MicroRNAs, and Myeloperoxidase, which have proved their value in several clinical trials (102). But more evidence is needed for them to be popularized and recognized.

### 4.2 Permissive cardiotoxicity

Cardiovascular clinical biomarkers help identify patients with cardiotoxicity. However, in the regulation for cardiac toxicity, it is insufficient to merely recognize patients with abnormal biomarkers. The challenge lies in balancing the preservation of cardiovascular health with the therapeutic benefits of cancer treatment. Premature intervention may result in unnecessary discontinuation of oncological therapy, while delayed intervention may result in significant cardiovascular damage. Both scenarios ultimately compromise the patient’s maximal benefit. This issue introduces the concept of “permissive cardiotoxicity”, which was first proposed by Charles Porter in 2022 (110). The concept aims to address the critical question “when to intervene”.

In their study, Porter et al. categorized cardiac toxicity into asymptomatic cardiotoxicity, mild to moderate cardiotoxicity, and life-threatening cardiotoxicity (110). For cardiotoxicity induced by HER-2 targeted therapies, they suggested intervention in the following four conditions: (a) a reduction in left ventricular ejection fraction (LVEF) ≥10% with an absolute LVEF <50%, (b) a 15% decrease in global longitudinal strain (GLS) from baseline, (c) elevation of BNP or troponin levels, and (d) symptomatic HF. Shijie Zhou et al. conducted follow-up on 51 patients treated with trastuzumab who developed permissive cardiotoxicity (111). The majority of these patients (92%) were able to complete their trastuzumab regimen, thereby validating the concept. However, 6% of patients were forced to discontinue treatment due to severe left heart dysfunction or HF, suggesting that further optimization of intervention timing is necessary. Porter et al. also noted that patients with pre-existing cardiovascular conditions have a narrower window for tolerable cardiac toxicity compared to the general population. The concept is a big step forward, but more trials are needed to establish tolerable cardiotoxicity criteria for different patients.

### 4.3 Status of targeted drug cardiotoxicity regulation

We must evaluate whether the three drugs in **Figure 1**’s red zone—trastuzumab, lapatinib, and T-DM1—require regulation. For trastuzumab, this is unequivocal. Large clinical trials around 2000 demonstrated its high efficacy, prompting the establishment of the Cardiac Review and Evaluation Committee (CREC) at Memorial Sloan-Kettering Cancer Center (13). In 2002, the CREC published criteria for trastuzumab-related cardiac dysfunction (CD), including LVEF decline, CHF symptoms, and related signs. Their analysis revealed a 27% incidence of CD with concurrent use of trastuzumab and anthracyclines, leading to the current practice of sequential administration. The CREC concluded that while trastuzumab’s cardiotoxicity is evident, it is acceptable given its therapeutic benefits. The FDA recommends regular LVEF assessments during therapy, with guidelines for discontinuation and resumption based on LVEF changes (112).

Lapatinib is frequently combined with trastuzumab. A meta-analysis of dual anti-HER-2 therapy indicates that this combination does not increase cardiotoxicity risk (18). The FDA recommends assessing LVEF before and during lapatinib therapy, with immediate discontinuation if LVEF falls below the institution’s LLN. If LVEF normalizes after two weeks, therapy may resume at a reduced dose (113).

T-DM1 is generally considered less cardiotoxic than trastuzumab, but cardiac monitoring remains essential (114, 115). The FDA advises LVEF evaluation before and during T-DM1 treatment. Specific dose adjustments are recommended based on LVEF levels: discontinue if LVEF is <40%, suspend and reassess if LVEF is 40-45% with a >10% reduction, and continue treatment if the reduction is <10% (116).

Recently, cardio-oncology guidelines were published in Europe and China (117, 118), both recommending similar management processes: assessment, monitoring, and follow-up. If cardiotoxicity is detected during monitoring or follow-up, treatment is initiated. The assessment phase considers various risk factors. European guidelines provide a specialized cardiovascular risk stratification table for HER-2 targeted therapy, categorizing patients into four levels: low, medium (subdivided into M1 and M2), high, and very high risk. The Chinese guidelines offer a universal stratification table with three levels: low, medium, and high risk. Both guidelines recommend baseline TTE and serum biomarker (cTn or NPs) measurements before therapy, with follow-up every three months during treatment. High and very high-risk patients are monitored at 3 and 12 months after treatment. More frequent assessments are conducted if deemed necessary by the clinician.

## 5 Conclusion

Breast cancer, excluding triple-negative cases, has a favorable prognosis with relatively low mortality. Targeted anti-HER-2 therapies have improved survival rates but introduced concerns about cardiotoxicity. Our review categorizes targeted drugs into red, yellow, and green based on cardiotoxicity risk, focusing on trastuzumab, lapatinib, and T-DM1 from the red category.

**Figure 2:**
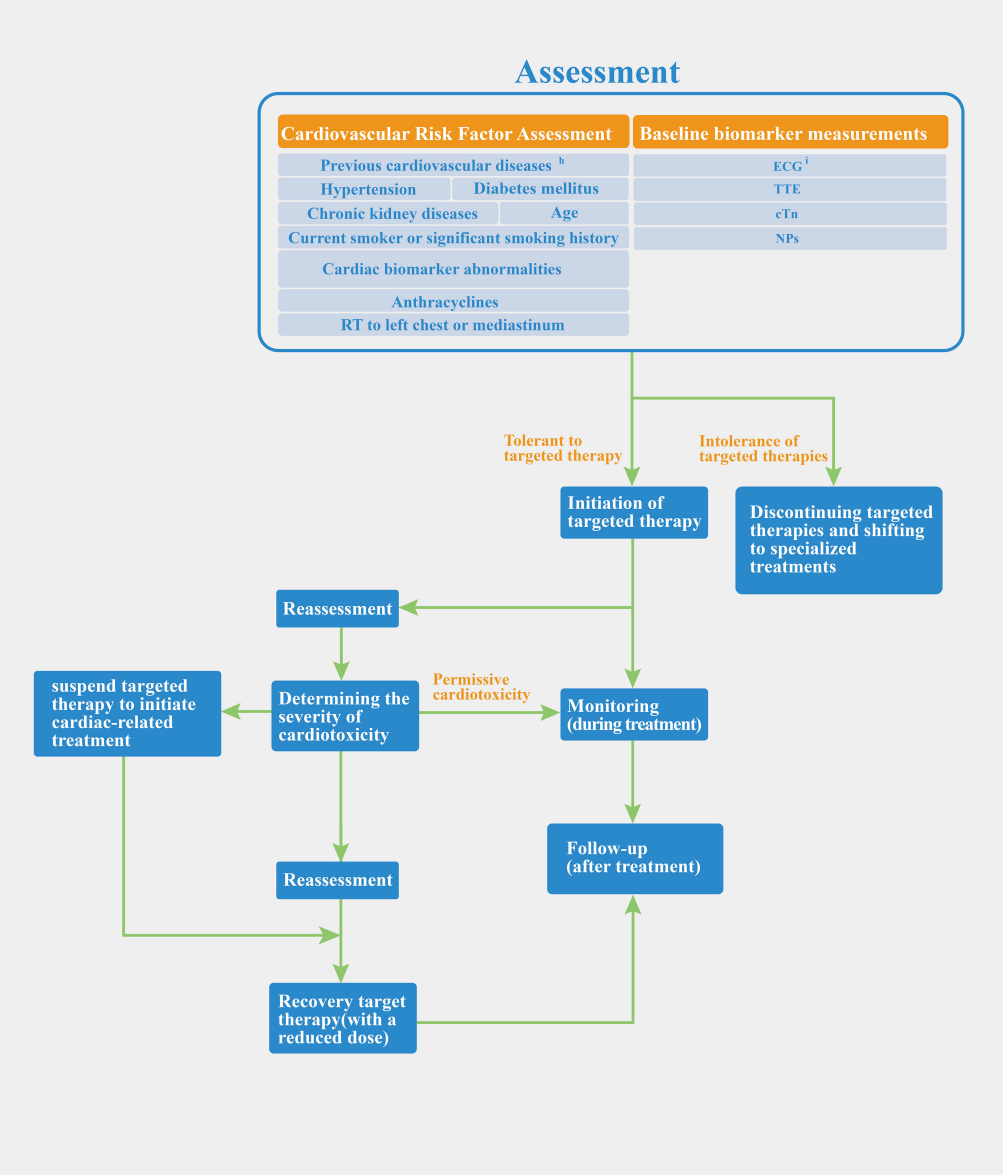
Preliminary regulatory process of anti-HER-2 targeted therapy. ^h^coronary heart disease, myocardial infarction, heart failure, cardiomyopathy, severe heart valve disease, arrhythmia; ^i^Chinese and European guidelines have different views in monitoring ECG. Chinese guidelines suggest that baseline measurements should be taken before the start of treatment. ECG should be performed during each treatment cycle and be monitored during the follow-up phase after the end of treatment. The European guidelines emphasize only baseline measurements before starting.

We discussed the common clinical biomarkers of cardiotoxicity and compared regulatory approaches. The concept of permissive cardiotoxicity was introduced to address the question of when to intervene in patients with cardiotoxicity. The FDA focuses on managing post-occurrence toxicity, while European/Chinese guidelines offer detailed protocols for evaluation and monitoring but lack specific dosage adjustment recommendations. We propose a preliminary regulatory process that integrates European and Chinese guidelines with FDA labeling, covering patient assessment, monitoring, and treatment adjustments. **Figure 2** illustrates this process, from baseline evaluation and risk stratification to treatment initiation, monitoring, and reassessment based on biomarker changes.

Current research and regulation of cardiotoxicity in targeted therapies have limitations, particularly regarding late cardiotoxicity, which is underexplored. This gap can be attributed to two factors: a shift in research priorities towards post-treatment tumor monitoring, with a historical focus on treatment efficacy and patient survival, and the significant improvement in survival rates for HER2-positive patients with anti-HER2 therapy. As a result, long-term cardiotoxicity has become more important, especially as older breast cancer survivors face greater cardiovascular risks than the likelihood of tumor recurrence (17). As research focus transitions from oncology treatments to long-term advantages for breast cancer survivors, the concern of late cardiotoxicity becomes prominent. Additionally, the duration required for extended follow-up is an important consideration. The absence of comprehensive long-term follow-up studies becomes increasingly pronounced as a drug is administered later in clinical practice. In the aforementioned Bradshaw PT article, a cohort of patients diagnosed with breast cancer was chosen (1996.8.1-1997.7.31) but the analysis did not encompass trastuzumab, the earliest FDA-approved anti-HER2-targeted drug, as it was approved subsequent to the study period (6). The later a drug is approved, the later it has to undergo late cardiotoxicity studies. Efforts are underway to standardize the definition of cardiotoxicity through guidelines and consensus from various regions. To address the second and third questions, we propose large, long-term retrospective studies to better capture the true incidence and understand long-term cardiotoxicity

Research into drug cardiotoxicity, especially its epidemiology, forms the basis for regulatory measures. Any gaps in this research can affect regulatory strategies. For example, if retrospective analysis reveals a higher incidence of cardiotoxicity with drugs like lapatinib and T-DM1 compared to RCT data, regulatory approaches must be adjusted accordingly. The current follow-up protocol involves monitoring high-risk individuals at three and twelve months after treatment completion (117, 118). However, if we gain a better understanding of the epidemiological characteristics of long-term cardiotoxicity associated with anti-HER-2-targeted medications, the follow-up strategy should be adjusted accordingly. Although a preliminary flowchart of the regulatory process has been provided, further refinement is necessary to address specific details. Specifically, the significance of serum biomarkers such as cTn and NPs in surveillance is widely acknowledged. However, there is a lack of consensus regarding the threshold at which their elevation necessitates the suspension of treatment or interruption of the intended goal. The guidelines suggest that monitoring frequency may be augmented for high-risk populations during treatment; however, further investigation into the cardiotoxicity of anti-HER-2 targeted medications is necessary to ascertain the extent of this increase. Ultimately, an accurate comprehension of cardiotoxicity is imperative for formulating an appropriate regulatory approach.
